# Mode of Action Analyses of Neferine, a Bisbenzylisoquinoline Alkaloid of Lotus (*Nelumbo nucifera*) against Multidrug-Resistant Tumor Cells

**DOI:** 10.3389/fphar.2017.00238

**Published:** 2017-05-05

**Authors:** Onat Kadioglu, Betty Y. K. Law, Simon W. F. Mok, Su-Wei Xu, Thomas Efferth, Vincent K. W. Wong

**Affiliations:** ^1^Department of Pharmaceutical Biology, Institute of Pharmacy and Biochemistry, University of MainzMainz, Germany; ^2^State Key Laboratory of Quality Research in Chinese Medicine, Macau University of Science and TechnologyMacau, China

**Keywords:** cancer, chemotherapy, drug resistance, natural products, neferine, P-glycoprotein

## Abstract

Neferine, a bisbenzylisoquinoline alkaloid isolated from the green seed embryos of Lotus (*Nelumbo nucifera* Gaertn), has been previously shown to have various anti-cancer effects. In the present study, we evaluated the effect of neferine in terms of P-glycoprotein (P-gp) inhibition via *in vitro* cytotoxicity assays, R123 uptake assays in drug-resistant cancer cells, *in silico* molecular docking analysis on human P-gp and *in silico* absorption, distribution, metabolism, and excretion (ADME), quantitative structure activity relationships (QSAR) and toxicity analyses. Lipinski rule of five were mainly considered for the ADME evaluation and the preset descriptors including number of hydrogen bond donor, acceptor, hERG IC_50_, logp, logD were considered for the QSAR analyses. Neferine revealed higher toxicity toward paclitaxel- and doxorubicin-resistant breast, lung or colon cancer cells, implying collateral sensitivity of these cells toward neferine. Increased R123 uptake was observed in a comparable manner to the control P-gp inhibitor, verapamil. Molecular docking analyses revealed that neferine still interacts with P-gp, even if R123 was pre-bound. Bioinformatical ADME and toxicity analyses revealed that neferine possesses the druggability parameters with no predicted toxicity. In conclusion, neferine may allocate the P-gp drug-binding pocket and prevent R123 binding in agreement with P-gp inhibition experiments, where neferine increased R123 uptake.

## Introduction

The main characteristics of cancer are uncontrolled growth and metastasis of mutated cells ultimately leading to the death of patients (Stratton et al., 2009). The World Health Organization (WHO) estimated around 8.2 million deaths and 14.1 million newly diagnosed cancer cases in 2012 worldwide. According to American Cancer Society reports, 1,688,780 new cancer cases and 600,920 cancer deaths are projected to occur in the United States in Siegel et al. (2017). Remarkably, underdeveloped countries had the largest incidence of new cases (8.0 million) and the highest death rates (5.3 million). Lifestyle risk factors such as smoking, obesity and physical inactivity will probably cause a further increase in cancer cases in the future (Jemal et al., 2011).

Chemotherapy is one of the mainstays of cancer management, often performed by simultaneously administering combination regimens of various agents. However, the success rate is limited due to drug resistance of tumor cells and high treatment-related toxicities (Jabbour et al., 2009; Eghtedar et al., 2013). Owing to severe side effects, chemotherapeutic drugs cannot be administered in doses high enough to reliably eliminate all tumor cells in the body, leading to narrow therapeutic indices.

In addition to severe toxicity, MDR represents a main problem of chemotherapy failure. MDR is characterized by cross-resistance to many structurally and mechanistically unrelated anticancer drugs (Gottesman and Ling, 2006; Gillet et al., 2007; Kuete et al., 2015). P-glycoprotein (P-gp) encoded by the *ABCB1/MDR1* gene is upregulated in many clinically resistant and refractory tumors and is referred as one of the most important mechanism of MDR (Kuete et al., 2015). Overexpression of P-glycoprotein (P-gp) is linked to accelerated efflux of chemotherapeutic agents. This process is fueled by ATP as energy source to perform the drug efflux. Targeting P-gp and other efflux pumps of the ABC transporter family represents a promising strategy to revert MDR and increase the efficiency of chemotherapy drugs (Abdelfatah and Efferth, 2015). The elucidation of the crystal structure of murine P-gp (Aller et al., 2009) and the development of homology models of human P-gp based on the murine amino acid structure have yielded valuable information to better understand poly-specific drug-binding in multiple conformations of P-gp (Aller et al., 2009; Zeino et al., 2014; Kadioglu et al., 2016).

In an endeavor to develop novel drugs with higher tumor specificity and lower toxicity to normal tissues, natural products serve as valuable source (Newman and Cragg, 2012; Cragg and Newman, 2013). Biosynthesis of natural products depends either on evolutionary adaption to the surrounding environments or as defense mechanism for the survival of organisms (Maplestone et al., 1992; Efferth et al., 2007). They can be of plant, animal, marine, or microorganismic origin. The bioactivity of natural products is based on the assumption that they possess specific activity toward different target proteins (Venkatraman, 2010) The challenging task of pharmacology is to identify disease-related targets that are relevant for therapeutic intervention in human patients (Cragg and Newman, 2013). Around 75% of approved anticancer agents are indeed based on natural products (Newman and Cragg, 2012; Cragg and Newman, 2013), which is a strong hint that natural products represent a promising reservoir for drug development.

Neferine is a bisbenzylisoquinoline alkaloid isolated from the green seed embryos of Lotus (*Nelumbo nucifera* Gaertn). This plant has been consumed in India and China since ancient times. In traditional Chinese medicine, it has been widely used to treat nervous disorders, insomnia, high fevers with restlessness, as well as pulmonary and cardiovascular diseases such as hypertension, atherosclerosis, restenosis, and arrhythmia (Sridhar and Bhat, 2007; Jun et al., 2016; Sharma et al., 2017). Various studies pointed out the potential anti-cancer effect of neferine as well (Huang et al., 2011b; Yoon et al., 2013; Poornima et al., 2014; Xu et al., 2016). In this study, we evaluated the effect of neferine in terms of P-gp inhibition via *in silico* molecular docking, QSAR and *in vitro* cytotoxicity assays and P-gp substrate uptake assays in drug-resistant cancer cells.

## Materials and Methods

### Cell Culture

All cells were obtained from the American Type Culture Collection (Rockville, MD, USA) unless otherwise specified. Taxol and doxorubicin-resistant types of MCF-7, A549 and HCT-8 cancers cells were purchased from KeyGEN BioTECH, China. All media were supplemented with 10% fetal bovine serum and the antibiotics penicillin (50 U/ml) and streptomycin (50 μg/ml; Invitrogen, Paisley, Scotland, UK). All cell cultures were incubated at 37°C in a 5% humidified CO_2_ incubator.

### Cytotoxicity Assays

Neferine was dissolved in DMSO at final concentrations of 100 mmol/L and stored at -20°C before use. Cytotoxicity was assessed using the 3-(4, 5-dimethylthiazol-2-yl)-2, 5-diphenyltetrazolium bromide (MTT) (5.0 mg/ml) assay. Briefly, 4 × 10^3^ cells were seeded per well in 96-well plates before drug treatment. After overnight culture, the cells were then exposed to different concentrations of neferine (0.039–100 μmol/L) for 72 h. Cells without drug treatment were used as control. Subsequently, MTT (10 μL) was added to each well and incubated at 37°C for 4 h followed by the addition of 100 μL solubilization buffer (10% SDS in 0.01 mol/L HCl) and overnight incubation. A_570 nm_ was determined from each well on the next day. The percentage of cell viability was calculated using the following formula: Cell viability (%) = A_treated_/A_control_ × 100. Data were obtained from three independent triplication experiments. Mean represented as ± SD. The IC50 value is directly obtained from the intercept between the growth curve and a horizontal line at 50% of viability in prism graph.

### Molecular Docking

The previously generated homology model of human P-gp (Tajima et al., 2014) was used for molecular docking studies with AutoDock 4 (Morris et al., 2009) on the drug-binding pocket. The residues at the drug-binding pocket were His61, Gly64, Leu65, Met69, Ser222, Leu304, Ile306,Tyr307, Phe336, Leu339, Ile340, Ala342, Phe343, Gln725, Phe728, Phe732, Leu762, Thr837, Ile868, Gly872, Phe942, Thr945, Tyr953, Leu975, Phe978, Ser979, Val982, Gly984, Ala985, Met986, Gly989, Gln990, and Ser993 (Aller et al., 2009). A grid map was chosen to cover these residues. Three independent docking calculations for neferine and R123 were conducted with 2,500,000 evaluations and 250 runs using Lamarckian Genetic Algorithm. The LBEs and predicted inhibition constants were obtained from the docking log files (dlg) and mean ± SD values were calculated. For visualization of the docking results, visual molecular dynamics (VMDs) were used. VMD software was developed with NIH support by the Theoretical and Computational Biophysics group at the Beckman Institute, University of Illinois at Urbana-Champaign. For co-docking calculations, R123 and neferine were selected to evaluate the effect of pre-docked compound on binding energies and docking pose.

### QSAR and Toxicity Prediction

Stardrop software (Optibrium, Cambridge, UK) was used to evaluate the druggability properties and toxicity of neferine. The ADME QSAR module of Stardrop was used to predict the druggability properties whereas the Derek Nexus module was used to predict the toxicities. ADME QSAR module helps to predict a broad range of ADME and physicochemical properties using a suite of high-quality QSAR models. Derek Nexus module involves a knowledge-based prediction of key toxicities by using data from published and unpublished sources. Derek Nexus identifies structure-toxicity relationships of compounds predicting the potential for toxicity. It calculated the likelihood of a compound causing toxicity based on more than 40 endpoints, including mutagenicity, hepatotoxicity, and cardiotoxicity.

The outputs from ADME QSAR and Derek Nexus modules were compared with that of verapamil. HBD, HBA, MW, logP, logD, TPSA, rotatable bonds, HIA, hERG (potassium channel) IC_50_ were selected as druggability descriptors. hERG activitiy is crucial for cardiac action potentials and blockade of hERG is linked with cardiac arrhythmia and cardiotoxicity. The parameters meeting the druggability properties were labeled green (H-bond donor < 5, H-bond acceptor < 10, MW < 500, logP < 5, logD < 5, TPSA < 140, Rotatable bonds < 10) (Veber, 2003; Lipinski et al., 2012). Plausible toxicities were labeled as pink, equivocal toxicities were labeled as yellow, inactive toxicities were labeled as green.

### Rhodamine 123 Exclusion Assay

The rhodamine 123 exclusion assay was carried out according to the reported procedures (Huang et al., 2012). Briefly, MDR cells were seeded in 6 well plate with 2 × 10^5^ cells each well and cultured for 24 h at 37°C in an atmosphere containing 5% CO_2_. At confluence, MDR cells were incubated with or without the modulator (10 μM of verapamil) and drug (1, 2, 5, and 10 μM of neferine) for 4 h at 37°C. Subsequently, 5 μg/mL of R123 was added to each well and the wells were incubated for another 1 h at 37°C. The accumulation of R123 was stopped by washing the cells five times with ice-cold PBS. The cells were then resuspended in 400 μL PBS for flow cytometry analysis. Intracellular fluorescence was measured using a flow cytometer at an excitation wavelength of 488 nm and emission wavelength of 525 nm. All data acquisition and analysis were performed with CellQuest (BD Biosciences, San Jose, CA, USA) with at least three independent experiments. Results were shown as the mean of fluorescence intensity.

### Statistical Analysis

The results were expressed as means ± SD as indicated. The difference was considered statistically significant when the *p*-value was less than 0.05. Student’s *t*-test analysis was used for comparison among different groups.

## Results

### Cytotoxicity Assay

**FIGURE 1:**
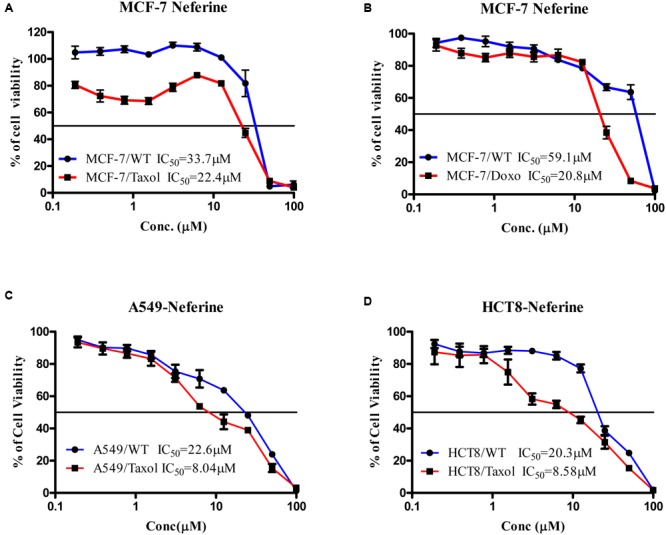
**Cytotoxicity of neferine toward paclitaxel-resistant and sensitive MCF-7 (A)**, doxorubicin-resistant and sensitive MCF-7 **(B)**, paclitaxel-resistant and sensitive A549 **(C)**, paclitaxel-resistant and sensitive HCT8 **(D)**.

Neferine yielded IC_50_ values in the low micromolar range for both sensitive and resistant cell lines. Interestingly, the IC_50_ values for the resistant sub-lines were even lower than those of sensitive cell lines, a phenomenon which is known as collateral sensitivity (Saeed et al., 2013). The results are summarized in **Figure 1**.

### Flow Cytometry

**FIGURE 2:**
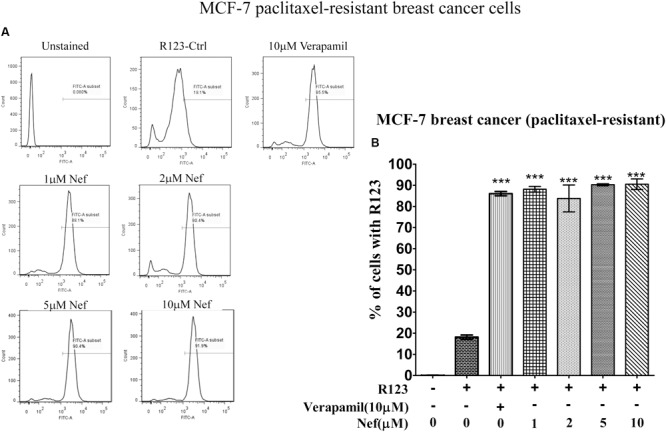
**R123 uptake assay of neferine on paclitaxel-resistant MCF-7 breast cancer cells. (A)** Represented the raw flow cytometry data of **(B)**, while **(B)** is the quantitative analysis of these flow cytometry data. ^∗∗∗^*p* < 0.001.

**FIGURE 3:**
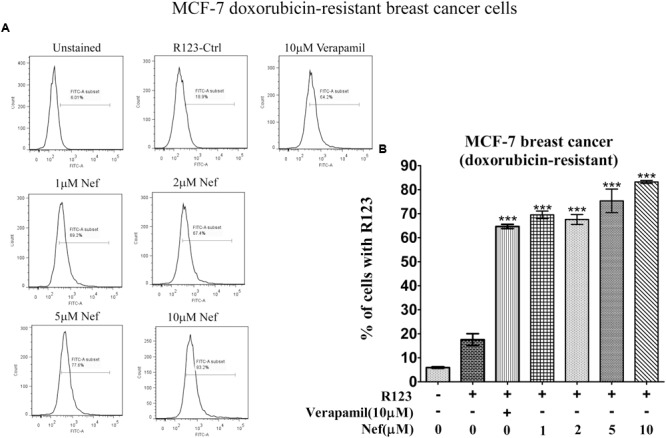
**R123 uptake assay of neferine on doxorubicin-resistant MCF-7 breast cancer cells. (A)** Represented the raw flow cytometry data of **(B)**, while **(B)** is the quantitative analysis of these flow cytometry data. ^∗∗∗^*p* < 0.001.

**FIGURE 4:**
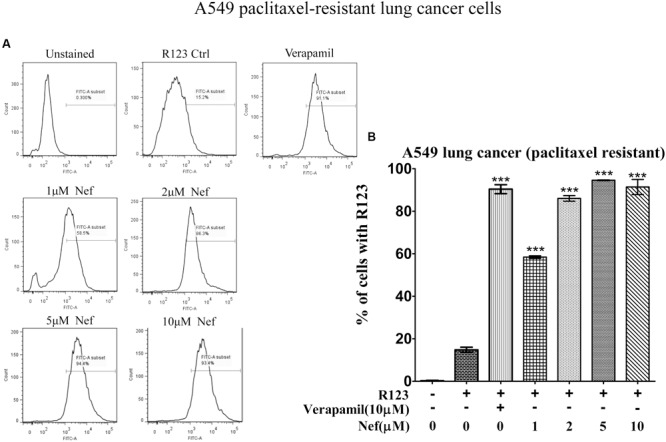
**R123 uptake assay of neferine on paclitaxel-resistant A549 lung cancer cells. (A)** Represented the raw flow cytometry data of **(B)**, while **(B)** is the quantitative analysis of these flow cytometry data. ^∗∗∗^*p* < 0.001.

**FIGURE 5:**
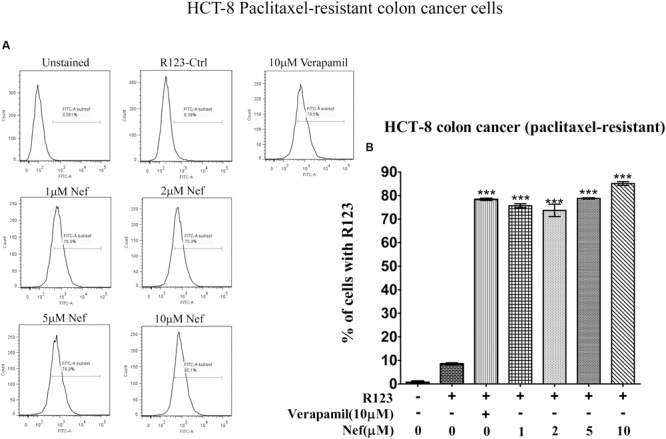
**R123 uptake assay of neferine on paclitaxel-resistant HCT8 colon cancer cells. (A)** Represented the raw flow cytometry data of **(B)**, while **(B)** is the quantitative analysis of these flow cytometry data. ^∗∗∗^*p* < 0.001.

Neferine increased R123 uptake in all drug-resistant cell lines (paclitaxel-resistant MCF-7, doxorubicin-resistant MCF-7 breast cancer cells, paclitaxel-resistant A549 lung cancer cells and paclitaxel-resistant HCT8 colon cancer cells) in a comparable manner, as the well-known P-gp inhibitor verapamil did. Even low concentrations (1 and 2 μM) yielded comparable increase in R123 uptake as verapamil (10 μM) in paclitaxel-resistant (**Figure 2**) or doxorubicin-resistant MCF-7 cells (**Figure 3**). In paclitaxel-resistant A549 cells, 2, 5, and 10 μM neferine revealed comparable increases of R123 than verapamil (**Figure 4**). Low neferine concentrations were enough to increase R123 uptake in paclitaxel-resistant HCT8 cells comparable to verapamil (**Figure 5**).

### Molecular Docking

**FIGURE 6:**
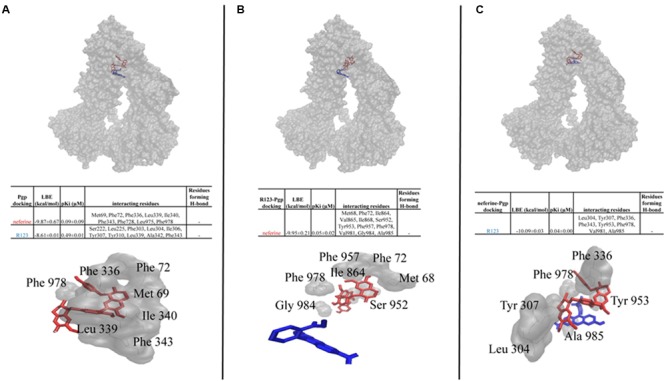
**Molecular docking analysis of neferine and R123 (A)**, neferine docking on R123 pre-bound human P-gp **(B)**, R123 docking on neferine pre-bound human P-gp **(C)**.

Neferine showed a similar docking pose and a higher LBE value (-9.64 ± 0.02 kcal/mol) than R123 (-8.61 ± 0.01 kcal/mol) on the drug-binding pocket of P-gp. In order to assess, whether pre-docking of R123/neferine could interfere with LBE values or docking poses, dockings of R123 and neferine to complexes of P-gp pre-docked with either of them were performed. If R123 was pre-docked, neferine still bound with high affinity (-8.62 ± 0.08 kcal/mol) on a site close to the drug-binding pocket. R123 docked to a neighboring site with lower affinity (-7.91 ± 0.01 kcal/mol), if neferine was bound on P-gp. These results clearly indicate that neferine docked to the drug-binding pocket with higher affinity than the known substrate R123. The results are summarized in **Figure 6**.

### QSAR and Toxicity Prediction

Bearing in mind that neferine would be pharmaceutically further developed as P-gp inhibitor, the question arises about the druggability properties and possible toxicity of this compound. Therefore, druggability and toxicities toward normal tissues for neferine were calculated by using the Stardrop software, via established QSAR model to evaluate the ADME properties and toxicities (**Table 1**). The established P-gp inhibitor, verapamil was used as positive control. Neferine achieved the preset, defined druggability properties (logS, logP, logD, HBD, HBA, TPSA, number of rotatable bonds) within the required limits as described in Section “Materials and Methods.” Values for those druggability properties were labeled bold in **Table 1**. Aqueous solubility was estimated by logS and lipophilicity was estimated by logP and logD. This implies that, neferine possess indeed the required druggability properties as it passed the majority of the ADME properties. However, neferine might cause skin sensitization, as this toxicity class was predicted as “equivocal.” Importantly, mutagenicity toxicity class was predicted to be absent. No other toxicities were indicated by the Stardrop software for neferine. Verapamil was predicted to possibly cause bladder disorders and hERG channel inhibition. Since the predicted IC_50_ value of neferine for hERG inhibition was higher than for verapamil, the risk of cardiotoxicity due to hERG inhibition is less probable for neferine than for verapamil.

**Table 1:** Druggability properties and toxicities of neferine and verapamil as predicted *in silico* by the Stardrop and Derek Nexus softwares.

	Neferine	Verapamil
logS @ pH7.4	**4.302**	**2.941**
logP	**4.432**	**3.790**
logD	**3.503**	**1.910**
2C9 pKi	5.736	5.315
hERG pIC50	7.814	6.155
BBB log([brain]:[blood])	–0.2118	0.35
BBB category	–	–
HIA category	+	+
P-gp category	Yes	Yes
2D6 affinity category	Low	Low
PPB90 category	Low	Low
MW	624.8	**454.6**
HBD	**1**	**0**
HBA	**8**	**6**
TPSA	**72.86**	**63.95**
Flexibility	0.1961	0.4118
Rotatable bonds	**10**	14
Photoallergenicity	N.R.	N.R.
Skin sensitization	**Equivocal**	N.R.
Occupational asthma	N.R.	N.R.
Respiratory sensitization	N.R.	N.R.
Developmental toxicity	N.R.	N.R.
Teratogenicity	N.R.	N.R.
Testicular toxicity	N.R.	N.R.
Adrenal gland toxicity	N.R.	N.R.
Hepatotoxicity	N.R.	N.R.
Ocular toxicity	N.R.	N.R.
Pulmonary toxicity	N.R.	N.R.
Splenotoxicity	N.R.	N.R.
Thyroid toxicity	N.R.	N.R.
Urolithiasis	N.R.	N.R.
Bladder disorders	N.R.	**Equivocal**
Bladder urothelial hyperplasia	N.R.	N.R.
Bone marrow toxicity	N.R.	N.R.
Cumulative effect on white cell count and immunology	N.R.	N.R.
Bradycardia	N.R.	N.R.
Cardiotoxicity	N.R.	N.R.
HERG channel inhibition	N.R.	**Plausible**
Kidney disorders	N.R.	N.R.
Kidney function-related toxicity	N.R.	N.R.
Nephrotoxicity	N.R.	N.R.
alpha-2-mu-Globulin nephropathy	N.R.	N.R.
Cholinesterase inhibition	N.R.	N.R.
Neurotoxicity	N.R.	N.R.
5 alpha-Reductase inhibition	N.R.	N.R.
Anaphylaxis	N.R.	N.R.
Blood in urine	N.R.	N.R.
Cerebral oedema	N.R.	N.R.
Chloracne	N.R.	N.R.
Cyanide-type effects	N.R.	N.R.
High acute toxicity	N.R.	N.R.
Methaemoglobinaemia	N.R.	N.R.
Estrogen receptor modulation	N.R.	N.R.
Estrogenicity	N.R.	N.R.
Peroxisome proliferation	N.R.	N.R.
Phospholipidosis	N.R.	N.R.
Phototoxicity	N.R.	N.R.
Mitochondrial dysfunction	N.R.	N.R.
Uncoupler of oxidative phosphorylation	N.R.	N.R.
Irritation (of the eye)	N.R.	N.R.
Irritation (of the gastrointestinal tract)	N.R.	N.R.
Irritation (of the respiratory tract)	N.R.	N.R.
Irritation (of the skin)	N.R.	N.R.
Lachrymation	N.R.	N.R.
Chromosome damage	N.R.	N.R.
Photo-induced chromosome damage	N.R.	N.R.
Mutagenicity	**Inactive**	**Inactive**
Photomutagenicity	N.R.	N.R.
Non-specific genotoxicity	N.R.	N.R.
Photo-induced non-specific genotoxicity	N.R.	N.R.
Carcinogenicity	N.R.	N.R.
Photocarcinogenicity	N.R.	N.R.

*Values for the druggability properties that meets the limits for the preset, defined druggability properties were labelled bolded. N.R., not reported.*

## Discussion

Neferine from green seed embryos of *Nelumbo nucifera* (Gaertn) is a bisbenzylisoquinoline alkaloid with cytotoxicity toward cancer cells (Poornima et al., 2014; Xu et al., 2016). It also revealed cell cycle arrest, apoptosis, endoplasmic reticulum stress, autophagy, as well as anti-angiogenic and anti-migratory effects in human hepatocellular carcinoma cells (Yoon et al., 2013). It also induces apoptosis in human lung cancer cells via the generation of reactive oxygen species (ROS), activation of MAPKs, lipid peroxidation, depletion of cellular antioxidant pools, loss of mitochondrial membrane potential, and intracellular calcium accumulation (Poornima et al., 2014). Another study reported that neferine synergistically reversed MDR in human gastric cancer cells (Huang et al., 2011b).

The present investigation aimed to analyze the inhibition by neferine of P-gp-mediated efflux of R123 in cancer cell lines insensitive to paclitaxel and doxorubicin treatments. R123 is a well-known substrate of P-gp (Kunihara et al., 1998) and its intracellular concentration is positively correlated to the degree of P-gp inhibition. We showed that neferine possessed P-gp inhibitory effect in all drug-resistant cell lines investigated. Of note, the drug-resistant cell lines exhibited collateral sensitivity (hypersensitivity) to neferine as well. Such P-gp-induced sensitivity of cancer cells, which are resistant to conventional chemotherapy, toward a new drug, is one of the proposed mechanisms against MDR (Pluchino et al., 2012; Saeed et al., 2013). One possibility is the generation of ROS due to excessive ATP consumption to export the drug by ABC transporters. Another P-gp-independent mechanism associated with the collateral sensitivity involves the alteration of membrane structure and fluidity which lead eventually to cancer cell death because of membrane perturbation (Bonavida, 2013; Saeed et al., 2013). Moreover, cancer cells primarily rely on glycolysis to meet energy needs, and have diminished rates of oxidative phosphorylation (the Warburg effect) (Cairns et al., 2011; Pluchino et al., 2012). Neferine increased the R123 accumulation in drug-resistant cells in a comparable manner with verapamil, which is a well-known P-gp inhibitor (Haq et al., 2001; Summers et al., 2004). Intriguingly, verapamil has also been shown to induce collateral sensitivity (Laberge et al., 2009; Hamm et al., 2014). P-gp inhibition was further supported by *in silico* molecular docking analysis by using a human P-gp homology model based on murine P-gp as template (Kadioglu et al., 2016). Since, the crystallographic structure of murine P-gp protein has been defined (Aller et al., 2009; Gutmann et al., 2010) and is sharing ∼87% identity to human P-gp in a drug-binding competent state (Saeed et al., 2015), our model well fit to the evaluation of binding energies and docking poses of neferine.

Molecular docking studies revealed that neferine binds to the drug-binding pocket with comparable binding energies to R123. In order to simulate molecular docking in a more realistic way to the functional experiments in cells, we recently introduced a co-docking approach for P-gp inhibitors at the drug-binding domain of P-gp (Kadioglu et al., 2016). This approach consists of two distinct phases of the interaction of substrates or inhibitors with P-gp: the first phase consists of the calculation of docking, if one compound interacts with P-gp and the second phase only takes place after P-gp interacted with another compound that is supposed to interfere with the first one. Interestingly, neferine strongly interacted with P-gp, even if R123 was pre-bound. This implies that neferine binds to the P-gp drug-binding domain without interference with R123 binding. As mentioned in **Figure 6**, neferine bound to another site within close proximity when R123 was pre-bound. Met68, Ile864, Val865, Ile868, Ser952, Tyr953, Phe957, Val981, Gly984, and Ala985 were the interacting amino acids in the molecular docking analyses on R123 pre-bound P-gp, which were different from the interacting amino acids for the molecular docking analyses on P-gp alone.

Numerous P-gp inhibitors have been described in the past decades (Bauer et al., 2005; Srinivas, 2016), but none of them has been approved for clinical use. This might be due to the fact that many P-gp inhibitors have not been specifically developed for this purpose, but are used for the treatment of other diseases. For example, verapamil is a calcium channel blocker employed to treat cardiovascular diseases (Haq et al., 2001; Summers et al., 2004). Therefore, novel P-gp inhibitors with higher specificity for P-gp alone are required. Considering that neferine revealed collateral sensitivity toward drug-resistant cancer cells in a comparable manner to verapamil as demonstrated by the R123 uptake experiment and that neferine interacted *in silico* with human P-gp strongly, even if R123 was pre-bound, neferine indeed possesses the potential as a promising drug candidate to fight against MDR. In fact, the druggability properties supported this hypothesis, since neferine met most druggability parameters as evaluated via Stardrop software (Obrezanova et al., 2008; Chadwick and Segall, 2010; Obrezanova and Segall, 2010; Segall and Chadwick, 2011). Toxicity predictions further supported the druggability potential of neferine, as it did not reveal any toxicity except equivocal skin sensitization whereas verapamil revealed plausible hERG inhibition, which is an important off-target that must be avoided during drug development (Sanguinetti and Tristani-Firouzi, 2006). Extensions to Lipinski’s rule of five parameters (logD, TPSA and number of rotatable bonds) (Veber, 2003) were also satisfied by neferine implying that neferine might possess sufficient druggability properties.

## Conclusion

The hereto reported neferine-induced collateral sensitivity toward drug-resistant tumor cells and increased R123 uptake imply the potency of applying neferine to MDR cancers therapy which is in line with other similar *in vitro* findings. Different from previously reported, in which the neferine-induced rendering of P-gp activity is related to the downregulation of P-gp and/or expression of the multidrug resistance protein 1 gene (mdr-1) (Cao et al., 2004; Xiao et al., 2005; Qin et al., 2010, 2011; Huang et al., 2011a,b) we verified that the direct strong interaction between neferine and P-gp is also involved. In addition, our data further illustrated the precise docking sites between neferine and P-gp which facilitate accurate drug design and scaling down the scope of novel chemical candidates screening. Most importantly, our *in silico* safety evaluations of neferine indicated a favorable toxicity profile of the compound, which justifies more detailed pre-clinical studies and provided platform for further investigation concerning the therapeutic potential of clinically using neferine upon cancer therapy.

## Author Contributions

TE and VW: conceived the study. OK: performed the *in silico* experiments. OK and TE: wrote the manuscript. BL, SM, and S-WX: performed the *in vitro* experiments. All the authors read the manuscript.

## Conflict of Interest Statement

The authors declare that the research was conducted in the absence of any commercial or financial relationships that could be construed as a potential conflict of interest.

## Abbreviations

ABC: ATP-binding cassette
ADME: absorption, distribution, metabolism, excretion
HBA: hydrogen bond acceptor
HBD: hydrogen bond donor
hERG: human ether-a-go-go related gene
HIA: human intestinal absorption
IC_50_: concentration to inhibit cellular proliferation by 50%
LBE: lowest binding energy
MDR: multidrug resistance
MW: molecular weight
P-gp: P-glycoprotein
QSAR: quantitative structure activity relationship
R123: rhodamine123
TPSA: total polar surface area
